# Cultivating a *Glocal* Virtual Classroom for Planetary Health

**DOI:** 10.1002/puh2.70265

**Published:** 2026-07-30

**Authors:** Dianne Jaula Cunanan, Julia Addison, Camilla Alay Llamas, George S. Downward, Joyce L. Browne, Renzo R. Guinto

**Affiliations:** ^1^ Mapúa School of Medicine Mapúa University Makati the Philippines; ^2^ Department of Global Public Health and Bioethics, Julius Center for Health Sciences and Primary Care Universitair Medisch Centrum Utrecht, Utrecht University Utrecht the Netherlands; ^3^ Institute for Risk Assessment Sciences (IRAS) Utrecht University Utrecht the Netherlands; ^4^ Utrecht University Centre for Global Challenges (UGlobe), Faculty of Law, Economics, Governance and Organization (REBO) Utrecht University Utrecht the Netherlands; ^5^ SingHealth Duke‐NUS Global Health Institute, Duke‐NUS Medical School National University of Singapore Singapore Singapore; ^6^ Planetary and Global Health Program St. Luke's Medical Center College of Medicine‐William H. Quasha Memorial Quezon City the Philippines

**Keywords:** climate action, decolonization, equity, medical education, online learning, planetary health, transformative learning, transnational education

## Abstract

The growing worldwide challenges of climate change, environmental degradation with biodiversity loss, and systemic inequities call for accessible, transformative, and impactful planetary health education that embraces a global perspective. However, traditional international learning experiences favor those with financial advantages, limiting equitable access to (planetary health) knowledge, intercultural collaboration competencies, and leadership opportunities. To address this challenge, the University Medical Center (UMC) Utrecht in the Netherlands and St. Luke's Medical Center College of Medicine's Planetary and Global Health Program (PGHP) in the Philippines developed a joint online course on Planetary Health and Climate‐Resilient Health Systems, combining synchronous and asynchronous learning activities. This novel approach allowed for collaboration between Dutch and Filipino medical students, integrating international perspectives while providing an opportunity to solve local problems and offering equitable access to education. The course incorporated online modules, workshops, and mentorshipblending expertise from educators from the Netherlands and the Philippines. Through the course, participants engaged in intercultural teamwork, focusing on student‐identified planetary health priority issues such as eco‐anxiety, fast fashion, and healthcare waste management. This initiative illustrates the importance and feasibility of collaborative, *glocal* approaches to planetary health education, with learners gaining insights into localized solutions for global issues. It also showed how the values of decolonizing global health education and bidirectional knowledge exchange can turn intercultural learning into educational practice. Future iterations will expand the course to involve more countries, refine the use of interactive tools, address time‐zone challenges, and incorporate education research to further study the learning process and outcomes. Institutional support is essential to scale this educational model, ensuring planetary health education remains accessible and impactful.

## Graphical abstract

A collaborative online international learning course in planetary health connects medical students from the Netherlands and the Philippines to address global challenges through local action. Blending synchronous and asynchronous learning, the program fosters intercultural collaboration, equitable access, and student‐led solutions on issues such as eco‐anxiety and healthcare waste. This initiative demonstrates a scalable, decolonial, and inclusive model for planetary health education.

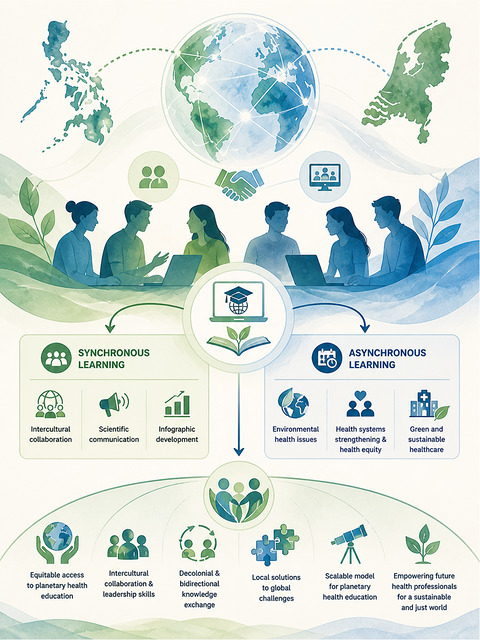

## 1 The Planetary Health Education Challenge

The field of planetary health, defined as “the health of human civilisation and the state of the natural systems on which it depends” [1], holds the potential to address the unsustainable relationship between people and the planet. To realize this potential, citizen education is essential to support the growing worldwide movement that calls for immediate transformative action to tackle the planetary health crisis.

As such, there is an urgent need for equitable, inclusive, and transformative education that invests in future leaders to tackle global and local challenges [2, 3]. In recent years, there has been a gradual rise in planetary health education, reflected in the development and use of the Planetary Health Education Framework [4], university programs [5], and innovative approaches such as massive open online courses [6], virtual academies [7], or field‐based learning [8]. Young people today demand more capacity‐building opportunities and leadership involvement in the evolving planetary health space [9]. Yet, there is a noticeable resource inequity—with most planetary health courses and initiatives created and/or offered in high‐income countries [5, 6]. Participation in globally oriented education activities often favors those with financial resources, limiting access for students in low‐resource settings and/or communities. This inequity is reflected in findings from the Planetary Health Report Card, which evaluated 188 institutions across 21 countries but included only a small number of schools from Africa, Asia, and South America, highlighting substantial geographic disparities in representation and access to planetary health education [3]. Online learning tackles this barrier and offers a more equitable alternative, unconstrained by geographic and financial limitations, with the potential to scale‐up and accommodate a larger number of students [10, 11].

## 2 A *Glocal* Virtual Classroom on Planetary Health

Planetary health challenges, while global in nature, manifest differently across communities and jurisdictions [6]. To address this, the University Medical Center (UMC) Utrecht in the Netherlands and St. Luke's Medical Center College of Medicine's Planetary and Global Health Program (PGHP) in the Philippines developed a joint “glocal” (global and local) virtual classroom to bring together learners from different socio‐cultural, economic, and health systems’ backgrounds.

**FIGURE 1:**
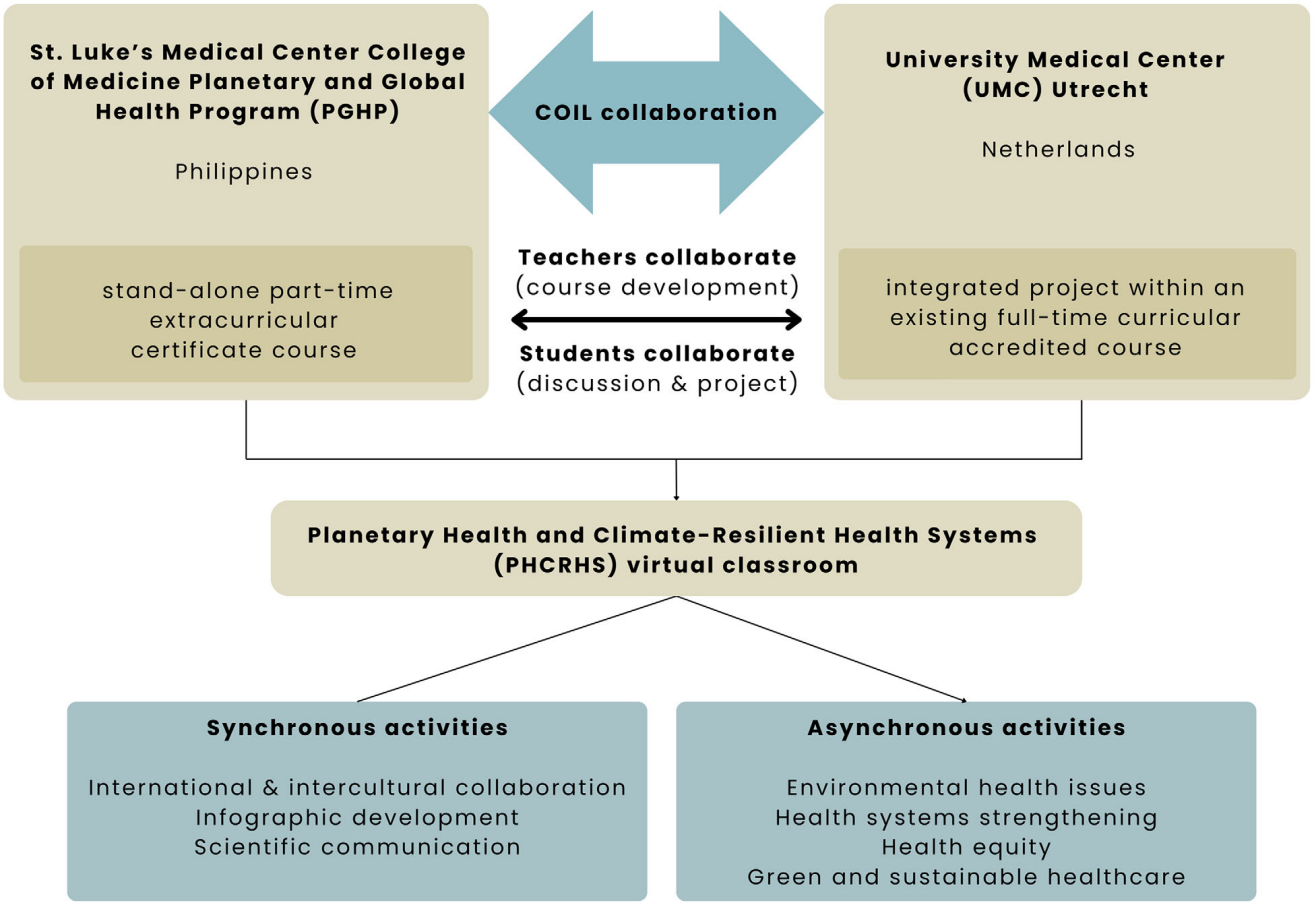
Structure of the Planetary Health and Climate‐Resilient Health Systems (PHCRHS) virtual classroom utilizing Collaborative Online International Learning (COIL) between the Philippines and the Netherlands.

The Collaborative Online International Learning (COIL) course “Planetary Health and Climate‐Resilient Health Systems (PHCRHS)” took place between February and April 2023 [10]. COIL is an innovative, didactic approach within the “internationalization at home” umbrella, which encompasses efforts to provide the benefits of international education into standard local curricula, connecting distant students and academics online for collaborative international learning [10]. Through this approach, the course aimed to not only equip students with planetary health knowledge but also to develop the transformative and intercultural competences necessary to enable them to become future agents of change in the field [2, 3]. Both synchronous and asynchronous learning activities were utilized toward this goal (Figure 1). Self‐paced online modules provide foundational knowledge on environmental health issues, health systems strengthening, health equity, and green and sustainable healthcare. Synchronous online workshops, blending Dutch and Filipino expertise, were dedicated to skills development in international and intercultural collaboration, infographic development, and scientific communication. Students worked together to design locally tailored solutions to global challenges, fostering an intercultural learning experience that challenges and provides an alternative to the inequitable flow of information often seen in modern‐day education and research [2].

The COIL component was structured to meet both institutions’ needs: a stand‐alone part‐time extracurricular certificate course for Filipino students and an integrated project within an existing full‐time curricular accredited course for Dutch students. The instructional team comprised educators from both countries. UMC Utrecht spearheaded logistics and the provision of online modules, whereas St. Luke's PGHP recruited Filipino students, provided administrative support, and offered in‐person mentorship. The course team aimed to reflect principles of justice and equity within the collaboration through explicit discussions on the distribution of workload, decision‐making, and benefits, equitable compensation for both teams, and shared representation in first and senior authorship of this commentary.

## 3 Student Experience and Feedback

The pilot course was a 12‐week collaborative online program co‐delivered between institutions in the Netherlands and the Philippines, designed as an initial implementation to evaluate feasibility, student engagement, and learning outcomes. During the pilot, eight Dutch and 15 Filipino students were grouped into four teams, collaborating on self‐identified topics such as eco‐anxiety, environmental impacts of fast fashion, healthcare waste management, and decolonizing global health.

A routine course evaluation was conducted, supplemented by an informal course review discussion held with students in which they expressed strong appreciation for the opportunity to collaborate with peers internationally. Students reported gaining deeper insights into planetary health issues and appreciated the varied local solutions required to address global problems.

**FIGURE 2:**
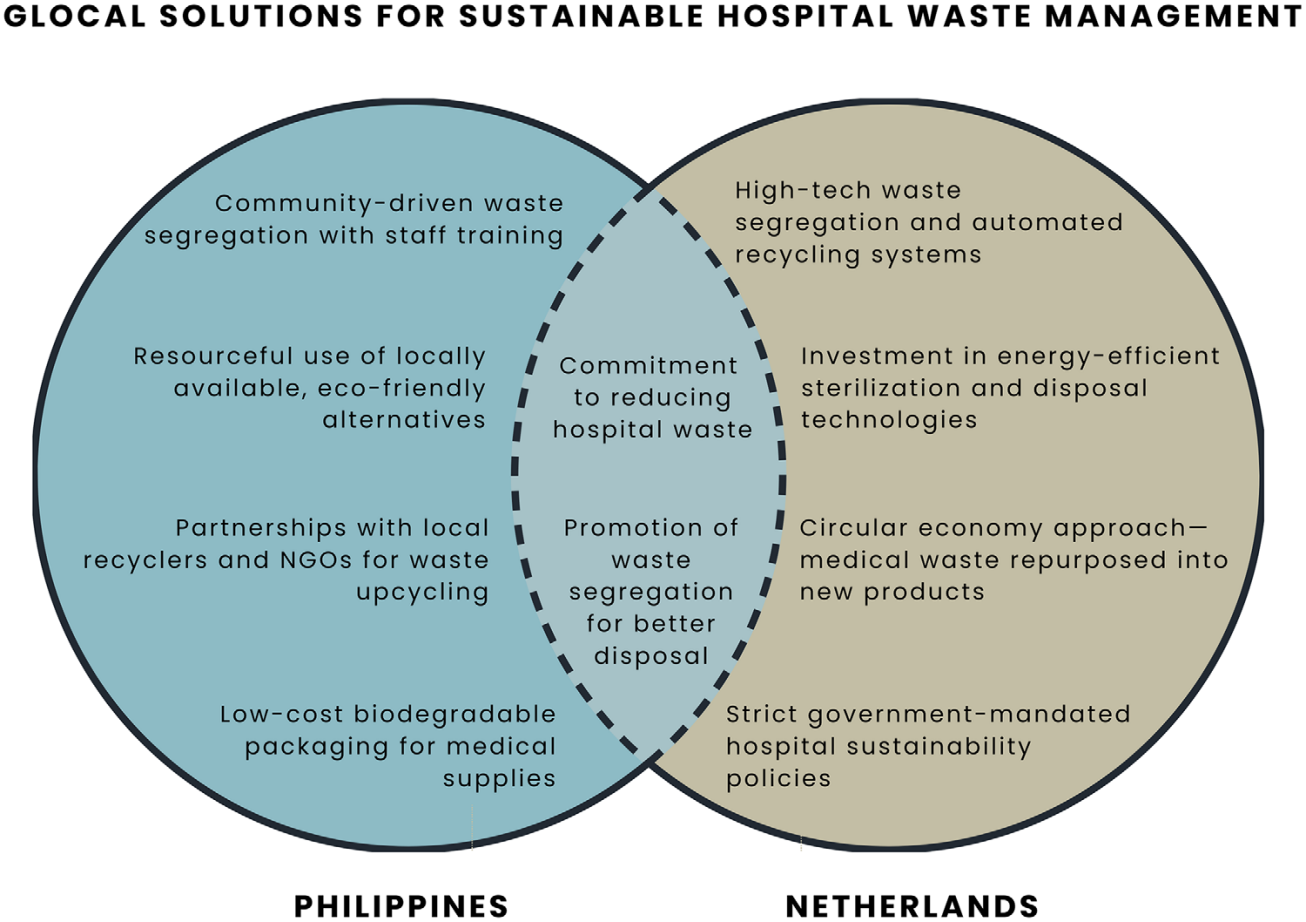
Venn diagram illustrating “glocal” solutions for sustainable hospital waste management, comparing approaches from the Philippines and the Netherlands, based on student presentations and discussions during the course. NGOs, non‐governmental organizations.

During course implementation, differences emerged in how students from each context experienced the course and approached problem‐solving. The final presentations demonstrated that students had indeed gained a nuanced and contextualized understanding of the *glocal* nature of planetary health problems and solutions. For example, although healthcare waste management is a global issue, proposed solutions vary by context. Dutch students more frequently emphasized systems‐level and technological solutions, such as optimizing recycling infrastructure, whereas Filipino students highlighted community‐based approaches, including decentralized waste management and local engagement strategies (Figure 2). Thus, this course also provided an experiential learning opportunity to understand what “just transition” means in the climate discourse—recognizing that each region has distinct economic and infrastructural constraints, necessitating tailored approaches to achieve environmental and social sustainability suitable for differing contexts [12].

Moreover, students noted that the course enabled them to experience bidirectional learning, countering the dominant unidirectional flow of knowledge that reflects ongoing colonial patterns in higher education, wherein the “expert” from the “North” unilaterally dictates dominant ideologies and perspectives [1, 3]. To help foster a safe online learning environment, the course began with an intercultural, international, and interdisciplinary collaboration workshop, setting the foundation for respectful and effective group work [10]. Through this approach, the course facilitated the exchange of diverse experiences and promoted collective understanding, which help challenge social prejudices and implicit biases.

The course design also provided an alternative model to conventional hierarchies in academia, contributing to efforts to decolonize planetary health education and foster equitable international collaborations [2]. This model can be adapted to other educational contexts, enabling institutions to promote *glocal* collaboration with greater inclusivity for learners from both the Global North and Global South. Additionally, the course introduced students to potential career paths in planetary health, including roles in education and medicine. Notably, the first author (D.J.C.) was a participant in this pilot course and now contributes to its succeeding editions as a staff member. Fittingly, these kinds of experiential learnings have been identified as an important didactic element of transformative education [2, 3].

## 4 Lessons Learned—And Limitations

The course's innovative design combined asynchronous and synchronous activities with a *glocal* approach, requiring students to develop context‐specific interventions for shared planetary health challenges. Such models are increasingly being adopted by universities exploring COIL [10, 11], underscoring their potential to promote equitable, sustainable, and impactful education.

This pilot course demonstrated the feasibility and effectiveness of a virtual, intercultural learning environment, offering important lessons for health educators worldwide. *Glocal* education models can break down barriers between high‐ and low‐resource contexts, promoting equitable access to knowledge, intercultural competency development, and leadership opportunities. Participating faculty expressed that the experience enhanced their competence in intercultural communication, international collaboration, and online teaching, as well as allowing them to learn from each other's distinct teaching approaches. This educational project also revealed to the teaching staff that virtual learning across time zones is feasible.

Although promising features and benefits of the pilot course were documented, several limitations were identified as well. In terms of the course itself, although it aimed to promote equitable collaboration, structural inequities between contexts may still have shaped group dynamics and access to learning opportunities. This study is limited by the small number of pilot participants and its focus on the first edition; two subsequent editions have since been conducted with broader country participation. Additionally, student feedback obtained was primarily qualitative in form and collected through routine evaluations rather than through a formal evaluation of an educational intervention. Further research using more robust evaluation methods is needed to assess its impact on student learning and leadership development.

## 5 Next Steps

Moving forward, to further promote this *glocal* virtual classroom, the spirit of co‐creation, coupled with a commitment to constant improvement, must be sustained. Suggestions for improvement include maximizing the use of interactive websites during lectures and providing weekly topic synthesis to allow the students more opportunities to connect and discuss the content covered in the online module. Future iterations could also expand the course to include more countries, facilitating broader intercultural collaboration. Time‐zone coordination and language diversity remain key challenges, requiring improvements in interactive tools and integration between asynchronous and synchronous content. Should more countries and cultures be included, one limitation is that English will likely remain the primary language of instruction. To mitigate this, the teaching team will strive to incorporate cultural and intellectual diversity into course content.

As educators continue to innovate, institutional support is essential to sustain and scale‐up promising educational modalities like this *glocal* virtual classroom. This institutional support should include encouragement to embed research into these educational innovations, to strengthen structured learning, and to support future scale‐up. Embedded research should include not only pre/post‐tests evaluating students’ international, intercultural, or transformative competencies to test the effectiveness of this education in reaching its intended goals, but also the competencies necessary for, and the effects of, offering COIL education on an institutional level [10]. Scale‐up should be further supported by faculty training (e.g., digital literacy, intercultural competence, implicit bias awareness, and international education) and administrative structures to support large‐scale sign‐ups of international students in online courses as well as open‐access platforms to host these courses that are not “owned” by one institute. By fostering equitable access to knowledge and leadership opportunities, such initiatives can empower learners and leaders in planetary health, regardless of their geographic or economic background. *Glocal* virtual classrooms can serve as blueprints for a new wave of educators seeking to develop creative, inclusive, and impactful programs that advance planetary health education in high‐ and low‐resource settings alike.

## Author Contributions

**Dianne Jaula Cunanan**: conceptualization, methodology, formal analysis, investigation, data curation, validation, visualization, writing – original draft, writing – review and editing, project administration. **Julia Addison**: conceptualization, methodology, formal analysis, investigation, data curation, validation, writing – original draft, writing – review and editing, project administration. **Camilla Alay Llamas**: methodology, validation, writing – review and editing, project administration. **George S. Downward**: methodology, validation, writing – review and editing. **Joyce L. Browne**: conceptualization, methodology, formal analysis, investigation, validation, writing – original draft, writing – review and editing, project administration, supervision, funding acquisition. **Renzo R. Guinto**: conceptualization, methodology, formal analysis, investigation, validation, writing – original draft, writing – review and editing, project administration, supervision. All authors have read and confirmed the final draft of the manuscript.

## Funding

This project has been funded by the Ministry of Education, Culture, and Science in the Netherlands and the Alliance between TU Eindhoven, Wageningen University & Research, Utrecht University, and UMC Utrecht (EWUU) in the Netherlands. The article processing charge was funded by Mapúa University.

## Conflicts of Interest

The authors declare no conflicts of interest.

## Data Availability

Data sharing is not applicable to this manuscript as no new data were generated, reported, or analyzed in this study.
